# Chirurgische Aufklärung: Klar geregelt durch das Patientenrechtegesetz – deutliche Unsicherheit bei Medizinstudierenden: Rechtsanalyse und Bestandsaufnahme bei über 2500 Berliner Medizinstudierenden im Rahmen des Progress Test Medizin

**DOI:** 10.1007/s00132-021-04080-1

**Published:** 2021-03-05

**Authors:** R. J. Seemann, P. Melcher, C. Eder, J. Deckena, R. Kasch, S. Fröhlich, M. März, AG Lehre DGOU, M. Ghanem

**Affiliations:** 1 Centrum für Muskuloskeletale Chirurgie, Charité – Universitätsmedizin Berlin, Augustenburger Platz 1, 13353 Berlin, Deutschland; 2 Klinik und Poliklinik für Orthopädie, Unfallchirurgie und Plastische Chirurgie, Universitätsklinikum Leipzig, Liebigstraße 20, 04103 Leipzig, Deutschland; 3 Geschäftsbereich Recht, Charité – Universitätsmedizin Berlin, Augustenburger Platz 1, 13353 Berlin, Deutschland; 4 Orthopädische Klinik und Poliklinik, Universitätsmedizin Rostock, Doberaner Str. 142, 18057 Rostock, Deutschland; 5 AG PTM, Charité – Universitätsmedizin Berlin, Charitéplatz 1, 10117 Berlin, Deutschland; 6 DGOU-Geschäftsstelle, Straße des 17. Juni 106–108, 10623 Berlin, Deutschland

**Keywords:** Co-Aktion, Aufklärungsgespräch, Medizinrecht, Berufliche Delegation, Medizinstudium, Co-Action, Informed consent, Medical jurisprudence, Professional delegation, Undergraduate medical education

## Abstract

**Hintergrund:**

Das chirurgische Aufklärungsgespräch stellt eine komplexe Herausforderung dar und ist als Lernziel im Nationalen Kompetenzbasierter Lernzielkatalog Medizin verankert. Die wenigen bestehenden Lehrformate sind uneinheitlich und aufwändig; insbesondere juristische Implikationen nehmen im Studium wenig Raum ein, obwohl sie mit Inkrafttreten des Patientenrechtegesetzes vermehrt in den Fokus gerückt sind und u. a. bei Regressverfahren eine wichtige Rolle spielen.

**Ziel der Arbeit:**

Ziel war eine Ermittlung des Wissensstandes von Medizinstudierenden zu den rechtlichen Aspekten des chirurgischen Aufklärungsgespräches auf Basis einer juristischen Analyse des Patientenrechtegesetzes. Dieses wurde auf Implikationen für die Lehre im Medizinstudium überprüft.

**Material und Methoden:**

Nach Analyse von Gesetz und Rechtsprechung wurden Multiple-Choice-Fragen zu den rechtlichen Aspekten des chirurgischen Aufklärungsgespräches erstellt und im Sinne einer Querschnittsanalyse im Progress Test Medizin platziert. Es erfolgte die deskriptive statistische Auswertung der Ergebnisse bei Berliner Medizinstudierenden.

**Ergebnisse:**

Es wurden die Antworten von 2625 (Wintersemester 2018/19) und 2409 (Sommersemester 2019) Berliner Studierenden ausgewertet. Bei den Fragen zur Art aufzuklärender Prozeduren sowie der Bedenkzeit nahm die Anzahl Studierender, die die Frage korrekt beantwortete, über die Zeit zu, erreichte jedoch nicht den Vergleichswert aller Fragen des Progress Test Medizin. Bei den Fragen zu den notwendigen Inhalten wählten, unabhängig vom Ausbildungsstand, zwischen 30 und 60 % die korrekte Antwort, eine Zunahme korrekter Antworten über die Zeit war nicht zu sehen.

**Diskussion:**

In der vorliegenden Arbeit konnte gezeigt werden, dass bei Medizinstudierenden über alle Semester hinweg Unsicherheiten bezüglich der juristischen Aspekte des Aufklärungsgespräches bestehen. Der gesetzliche Rahmen lässt allerdings Raum für neue Lehrformate wie der hier erstmals vorgestellten „Co-Aktion“: der Studierende führt die Aufklärung eines Patienten selbstständig, unter Aufsicht und Verantwortung des behandelnden Arztes, durch.

## Abstract (en)

**Background:**

Obtaining informed consent is a challenging task and is part of the educational objectives in the German NKLM. Teaching formats are inconsistent and time-consuming, with little emphasis on legal aspects, although they have moved into the focus of attention since the implementation of patient rights laws and play an important role in legal proceedings.

**Objectives:**

The aim of this study was the evaluation of medical students’ knowledge about the legal aspects of obtaining informed consent. A legal analysis was performed, and the patient rights laws were reviewed with reference to implications for undergraduate medical education.

**Materials and methods:**

After the analysis of laws and jurisdiction, multiple-choice questions regarding the legal aspects of obtaining informed consent were created and placed in the Progress Test Medicine (PTM). A statistical analysis of the results of Berlin medical students was performed descriptively.

**Results:**

The answers of 2625 (winter semester 2018/19) and 2409 (summer semester 2019) medical students in Berlin were analyzed. The rate of students who answered the questions about the procedures requiring informed consent and adequate time for consideration increased over time but did not reach comparable values to all PTM questions. Questions about required content were answered correctly by 30 to 60% of the students, regardless of their level of training; we did not see an increase along with the time of study.

**Conclusion:**

In our study, we were able to show that medical students of all educational levels show tentativeness when it comes to the legal aspects of obtaining informed consent. Yet, the legal framework offers room for new teaching formats like “Co-Action”, introduced in this paper for the first time, where students acquire informed consent while being supervised by the medical doctor in charge.

## Hintergrund und Fragestellung

Der Begriff „Informed Consent“ (dt.: „Informierte Einwilligung“) ist bereits seit den 1970er-Jahren in Medizin und Wissenschaft gebräuchlich und bezeichnet die durch vorausgehenden Informationsaustausch und ausführliche Aufklärung bedingte, freiwillige Zustimmung eines Patienten zu bestimmten (invasiven) medizinischen Maßnahmen [13]. Ein chirurgisches Aufklärungsgespräch stellt Ärztinnen und Ärzte dabei vor eine komplexe Herausforderung auf mehreren Ebenen: neben der *fachlich-inhaltlichen* und der *kommunikativen* – der oder die Aufklärende verfügt über das zur Erläuterung der bevorstehenden Maßnahme notwendige Fachwissen und kann sie einem medizinischen Laien verständlich und empathisch vermitteln – ist dies auch die *juristische* Ebene. Spätestens mit Inkrafttreten des Patientenrechtegesetzes §§630a ff. BGB im Jahr 2013 ist das Aufklärungsgespräch mit all seinen inhaltlichen und rechtlichen Implikationen vermehrt in den Fokus der öffentlichen Aufmerksamkeit gerückt.

Bereits in der universitären Ausbildung soll das Führen eines chirurgischen Aufklärungsgespräches Teil des Curriculums sein. Der Nationale Kompetenzbasierte Lernzielkatalog Medizin (NKLM) enthält das entsprechende Lernziel an Position 14c.2.8.6.: „ein Aufklärungsgespräch führen“ [12]. In den verschiedenen Fakultäten scheint das nicht immer umgesetzt zu sein (mündliche Mitteilung durch mehrere Lehrkoordinatoren). Im Modellstudiengang der Charité Berlin wird das Führen eines Aufklärungsgespräches zum einen im Rahmen eines Termins „Kommunikation-Interaktion-Teamtraining“ im 5. Semester [7] sowie in einem Vorlesungstermin direkt vor PJ-Beginn zum Thema „Arztrecht“ [6] gelehrt. Ein einheitliches Lehr- oder Prüfungsformat existiert bislang nicht; mögliche Ansätze umfassen aufwändige Unterrichtsmethoden wie beispielsweise die Arbeit mit Simulationspatienten und Videofeedback [17, 19]. Die juristische Ebene und die rechtlichen Rahmenbedingungen nehmen in diesen Ansätzen eine untergeordnete Rolle ein.

### Juristischer Hintergrund

Mit dem erklärten Zweck, Transparenz und Rechtssicherheit für die Patienten zu schaffen [8], wurden im Rahmen des Patientenrechtegesetzes in den §§630a ff. BGB zahlreiche Anforderungen an das Behandlungsverhältnis zwischen Arzt und Patient kodifiziert, die bis dato überwiegend von der Rechtsprechung entwickelt wurden. Einen Schwerpunkt bildet dabei die Regelung der Aufklärung in §630e BGB. Im Zuge der Kodifizierung wurden viele zuvor nicht näher geregelte Aufklärungsvoraussetzungen und Informationspflichten des Arztes festgelegt. Dazu zählen etwa der Umfang der Aufklärung (Abs. 1), die Person des Aufklärenden (Abs. 2 Nr. 1) und der angemessene Zeitpunkt (Abs. 2 Nr. 2). In der Praxis verbleiben dennoch Unsicherheiten: Über welche Risiken nach Absatz 1 ist genau aufzuklären? Was bedeutet es, wenn gemäß Absatz 2 Nr. 1 die Person des Aufklärenden die „notwendige Ausbildung“ dafür haben muss? Wann ist eine Aufklärung nach Absatz 2 Nr. 2 (noch) „rechtzeitig“?

### Fragestellung

Die vorliegende Arbeit integriert zwei Fragestellungen: Zunächst wurde eine juristische Analyse des Patientenrechtegesetzes durchgeführt und dieses auf die Implikationen für die Lehre im Medizinstudium überprüft. Die darauf aufbauende Querschnittsstudie hatte eine Ermittlung des Wissensstandes von Berliner Studierenden der Humanmedizin zu den rechtlichen Aspekten des chirurgischen Aufklärungsgespräches in Abhängigkeit ihres Fachsemesters zum Ziel.

## Studiendesign und Untersuchungsmethoden

### Der Progress Test Medizin (PTM)

Der PTM wurde an der Charité – Universitätsmedizin Berlin nach internationalem Vorbild [2, 20] entwickelt und ist ein fächerübergreifender, formativer Test für Medizinstudierende, der 200 MC-Fragen auf Absolventenniveau beinhaltet [15]. Seit 1999 wird er jedes Semester durchgeführt, zunächst an der Charité, mittlerweile an insgesamt 14 deutschen und österreichischen Fakultäten. Die Teilnahme ist für jeden Studierenden jedes Semester freiwillig und hat keinen Einfluss auf die Studienleistungsbewertung. Der PTM erlaubt Aussagen zu Wissensstand und Wissenszuwachs der Medizinstudierenden in den verschiedenen Fachbereichen oder ganz gezielt in Bezug auf einzelne Fragestellungen [14]. Als MC-Test eignet er sich für die Abfrage von Fachwissen in unterschiedlichen Teilaspekten des Curriculums (u. a. also auch medizinrechtliche Fragestellungen).

### Juristische Analyse und Erstellung von geeigneten MC-Fragen

Die rechtlichen Rahmenbedingungen und gesetzlichen Anforderungen, insbesondere nach den §§630a ff. BGB an das Aufklärungsgespräch wurden durch die Rechtsabteilung der Klinik untersucht und die wesentlichen Aussagen identifiziert. Dies stellte die Basis dar für die anschließende Erstellung von inhaltlich abgestimmten MC-Fragen nach den Richtlinien zur Erstellung qualitativ hochwertiger Fragen [1, 11] durch ein Expertenteam, bestehend aus wissenschaftlichen Mitarbeitern der AG Progress Test Medizin (PTM) Berlin und Mitgliedern der AG Lehre der Deutschen Gesellschaft für Orthopädie und Unfallchirurgie (DGOU). Eine Frage beschäftigte sich mit den aufzuklärenden Prozeduren, eine mit der angemessenen Bedenkzeit und zwei mit den notwendigen Inhalten eines Aufklärungsgespräches. Alle Fragen durchliefen einen mehrstufigen Review-Prozess, bevor sie in die Fragendatenbank des PTM aufgenommen und ab dem WS 2018/19 erstmals eingesetzt wurden.

### Platzierung der Fragen im PTM

Die vier Fragen wurden im WS2018/19 und SS2019 in den PTM platziert, Fragen 1–3a im WS2018/19, Frage 3b im SS2019. Im WS 2018/19 nahmen am PTM 39 in Berlin 3254 Studierende teil, durchschnittlich 319 (min: 282, max.: 353) Studierende pro Semester in den Semestern 1–10. Da sich die Studierenden im 11. Semester bereits im PJ befinden und eine Teilnahme am PTM nicht obligat ist, zeigt sich in dieser Gruppe erfahrungsgemäß eine deutlich reduzierte Teilnehmerzahl, sodass nur die Semester 1–10 ausgewertet wurden. Im SS2019 nahmen am PTM 40 in Berlin 3174 Studierende teil, mit durchschnittlich 312 (min: 274, max.: 345) Studierenden in den Semestern 1–10. In die Auswertung gingen nur die Antworten „ernsthafter“ Teilnehmer ein; Teilnehmer, die keine Frage beantwortet hatten oder deren Antwortmuster probabilistisch als „Raten“ eingestuft wurde, flossen nicht in die Auswertung ein. Im WS 2018/2019 waren dies durchschnittlich 263 Studierende (min: 232, max.: 298) und im SS 2019 241 Studierende (min: 197, max.: 292). Die Qualitätsmerkmale Schwierigkeit und Trennschärfe in Bezug auf die einzelnen Fragen sind in Tab. 1 aufgelistet. Die Schwierigkeit ist definiert als Quotient aus der Anzahl der Teilnehmer, die eine Frage korrekt beantworteten, und der Gesamtzahl von Teilnehmern. Die Trennschärfe ist definiert als Korrelationskoeffizient zwischen der Folge aller Antworten der Frage (hier wurde nur zwischen „richtig“ oder „nicht richtig“ unterschieden) und der Folge der Anzahl von richtigen Antworten pro Teilnehmer; gemessen wurde also, ob die Gruppe der Teilnehmer, die eine Frage richtig beantwortet hat, mit der Gruppe der leistungsstarken Teilnehmer übereinstimmt [11].

**Table 1:** 

Frage	Trennschärfe	Schwierigkeit
1	0,36	0,36
2	0,48	0,45
3a	0,28	0,47
3b	0,22	0,36

### Auswertung

Die deskriptive statistische Auswertung erfolgte mittels Excel (Fa. Microsoft Inc., Redmond, WA, USA). Es wurde das Antwortverhalten aller Studierenden analysiert sowie die Qualitätskriterien Item-Trennschärfe und Item-Schwierigkeit jeder Frage ermittelt [11].

## Ergebnisse

**Figure 1:**
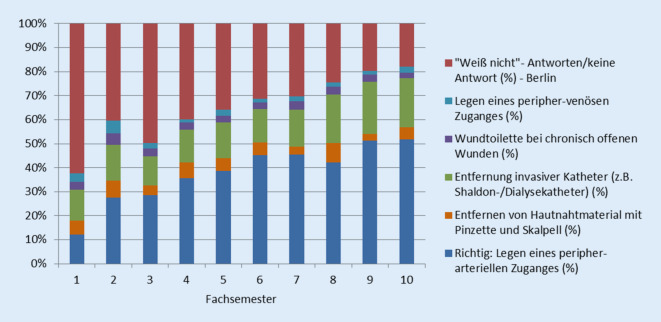

Frage 1 (Abb. 1) bot sechs Antwortmöglichkeiten a–f inklusive „Weiß nicht“ an. Im 1. Semester beantworteten im Durchschnitt 12,05 % der Studierenden diese Frage korrekt (blau), 62,25 % wählten „Weiß nicht“ oder keine Antwort (rot). Über die Zeit verschob sich dieses Verhältnis zugunsten der korrekten Antwort, sodass die Studierenden im 10. Semester zu 51,79 % die richtige Antwort wählten und 17,86 % „Weiß nicht“/keine Antwort. Als falsche Antwort bzw. Distraktor wurde Antwort c (grün) über alle Semester hinweg am häufigsten (min. 11,92 %, max. 21,81 %) ausgewählt.

**Figure 2:**
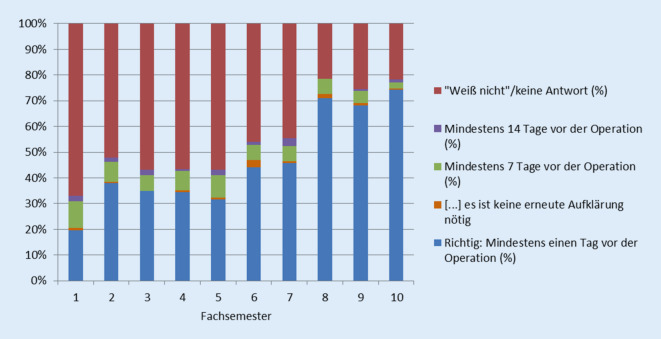

Frage 2 (Abb. 2) bot fünf Antwortmöglichkeiten a–e inklusive „Weiß nicht“ an. Wiederum zeigt sich hier ein Wissenszuwachs über die Zeit: im 1. Semester wählten im Durchschnitt 19,68 % der Studierenden die richtige Antwort (blau) aus, während 67,07 % „Weiß nicht“/keine Antwort (rot) wählten. Im 10. Semester wählten 71,43 % der Studierenden die richtige Antwort, 21,79 % „Weiß nicht“/keine Antwort. Antwort c (grün) wurde als Falschantwort am häufigsten (min. 2,5 %, max. 10,44 %) gewählt.

**Figure 3:**
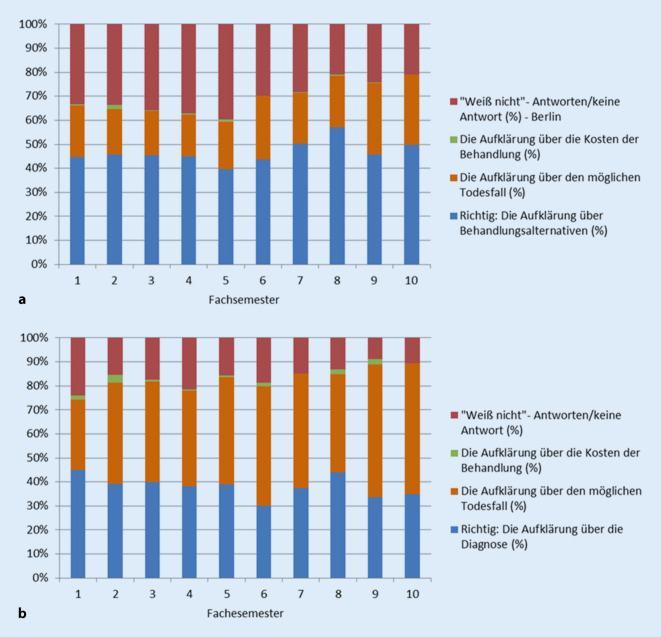

Frage 3a und 3b (Abb. 3) hatten denselben Wortlaut und identische Distraktoren, jedoch jeweils unterschiedliche richtige Antwortmöglichkeiten. Bei Frage 3a wurde die richtige Antwort (blau) im Schnitt von 46,63 % der Studierenden zwischen 1. und 10. Semester gewählt, „Weiß nicht“/keine Antwort (rot) wurde im 1. Semester von 33,33 %, im 10. Semester von 21,07 % gewählt. Die Falschantwort a (Aufklärung über den möglichen Todesfall, orange) wurde ebenfalls auffällig oft gewählt (min. 17,39 %, max. 29,87 %), in höheren Semestern häufiger als in niedrigen. Frage 3b wurde im Schnitt von 28,69 % der Studierenden zwischen 1. und 10. Semester richtig (blau) beantwortet. Im 1. Semester wählten mit 21,18 % die meisten Teilnehmer „Weiß nicht“/keine Antwort (rot), im 10. Semester waren es noch 8,56 %. Hier wurde der Distraktor a (Aufklärung über den möglichen Todesfall, orange) über fast alle Semester hinweg häufiger gewählt als die richtige Antwortmöglichkeit.

## Diskussion

In der vorliegenden Arbeit konnte gezeigt werden, dass bei Medizinstudierenden über alle Semester hinweg Unsicherheiten bezüglich der juristischen Aspekte des Themas „Aufklärungsgespräch“ bestehen, auch wenn die Fragen zum Ende der Studienzeit häufiger korrekt beantwortet wurden. Nach eingehender juristischer Analyse lässt der gesetzliche Rahmen allerdings Raum für alternative Lehrformate wie der hier erstmals vorgestellten „Co-Aktion“: Studierende führen in Anwesenheit und unter Aufsicht des behandelnden Arztes ein Aufklärungsgespräch selbstständig durch.

Die in vorliegender Studie untersuchten vier Fragen prüfen unterschiedliche Aspekte ab. Frage 1 beschäftigte sich mit aufklärungspflichtigen Eingriffen im medizinischen Alltag. Die Tatsache, dass der Anteil Studierender, die diese Frage korrekt beantworten konnte, über die Zeit zunahm, spiegelt möglicherweise dabei das zunehmende medizinische Grundverständnis und die zunehmende Praxiserfahrung wider. Ab dem 6. Semester kreuzten mehr Studierende die korrekte Antwort an als „Weiß nicht“ oder keine Antwort, was möglicherweise mit dem im Berliner Modellstudiengang im fünften Semester angesiedelten KIT(Kommunikation/Interaktion/Teamtraining)-Termin „Aufklärungsgespräch“ [7] zusammenhängt. Dennoch wusste nur gut die Hälfte der Teilnehmer im 10. Semester die korrekte Antwort, weniger als der Vergleichswert (60 %) aller Fragen zum Studienende.

Frage 2 behandelte die angemessene Bedenkzeit. Deutlicher als in Frage 1 nahm der Anteil korrekter Antworten von Studierenden über die Zeit zu, bis er im 10. Semester über 70 % betrug. Parallel dazu nahm der Anteil Studierender, der bei dieser Frage „Weiß nicht“/keine Antwort wählte, von mehr als 60 % im 1. Semester auf gut 20 % im 10. Semester ab. Auch hier spielen sicherlich zunehmendes medizinisches Grundverständnis und praktische Erfahrung eine Rolle. Zwischen dem 7. und 8. Semester ist ein deutlicher Sprung zu erkennen, der vermutlich mit der im Berliner Modellstudiengang im 7. Semester angesiedelten Lehre in chirurgischen Fächern zusammenhängt. Insgesamt wurde diese Frage zum Studienende hin überdurchschnittlich gut beantwortet. Dabei ist zu berücksichtigen, dass die Antwort gefestigter Rechtsprechung^1^ entspricht. Auch wenn sie nicht in den Gesetzeswortlaut formuliert wird, hat sie zumindest Eingang in die Gesetzesbegründung gefunden.^2^

Die Fragen 3a und 3b hatten die notwendigen Inhalte eines Aufklärungsgespräches zum Thema. Orientierend an unseren Untersuchungen bereiteten sie den Studierenden die meisten Schwierigkeiten. Über alle Semester hinweg wählten lediglich 40–50 % korrekterweise die „Behandlungsalternativen“, lediglich 18–30 % die „Diagnose“ als zum Aufklärungsgespräch am ehesten zugehörig aus. Eine mögliche Ursache dafür, dass viele Studierende die „Aufklärung über den möglichen Todesfall“ vor „Behandlungsalternativen“ und „Diagnose“ wählten, ist, dass sowohl die Diagnose, als auch mögliche Behandlungsalternativen und Risiken Inhalt der Selbstbestimmungsaufklärung sein können. Über den möglichen Todesfall ist grundsätzlich allerdings nur dann aufzuklären, wenn dieser ein für die Einwilligung in die Behandlung relevantes Risiko darstellt. Das wiederum ist eine Frage des Einzelfalls: Nicht bei jeder Behandlung besteht ein entsprechendes Risiko, über das aufzuklären wäre. Deshalb wurde die Frage um die Spezifikatoren „[…] im Normalfall am ehesten […]“ ergänzt, um keinen Raum für Interpretation zu lassen. Alles in allem scheint den Studierenden das Bewusstsein dafür zu fehlen, dass es im Aufklärungsgespräch nicht darum geht, jede erdenkliche Eventualität zu benennen, sondern der Patientin oder dem Patienten ein angemessenes Bild von der notwendigen Prozedur zu vermitteln.

Zur guten Leistungsdifferenzierung in einem MC-Test sind Items mit einer Schwierigkeit zwischen 0,4 und 0,9 gewünscht [10]. Der PTM hat die Besonderheit, dass er Wissen unabhängig vom tatsächlichen Wissenstand abprüft und dieselbe Frage Erst- wie auch Zehntsemestern gestellt wird, sodass die Schwierigkeit der Fragen tendenziell überschätzt wird. Die in dieser Studie verwendeten Fragen wiesen Schwierigkeiten zwischen 0,36 und 0,69 auf und bewegen sich damit in einem für den PTM akzeptablen Bereich (mittlere Schwierigkeit der PTM-Fragen über alle Semester 0,31). Die Trennschärfe einer Frage sollte über 0,2 liegen, um Leistung zuverlässig zu differenzieren [10]. Die in dieser Studie verwendeten Fragen wiesen Trennschärfen zwischen 0,22 und 0,48 auf (mittlere Trennschärfe der PTM-Fragen über alle Semester 0,37).

Chirurgische Fächer, allen voran Orthopädie und Unfallchirurgie, sind besonders exponiert, wenn es um Behandlungsfehler und Regressansprüche von Patienten geht [4, 5]. Obwohl dabei als wichtiger und direkt beeinflussbarer Faktor die umfassende und regelrechte, d. h. gesetzeskonforme, präoperative Aufklärung des Patienten identifiziert wurde [4], wird diese häufig von Assistenzärzten niedrigen Ausbildungsstandes durchgeführt [3], ohne dass zuvor eine systematische Schulung dazu stattgefunden hätte [9, 16, 18]. Auch die wirtschaftlichen Implikationen im Falle geltend gemachter Regressansprüche aufgrund fehlerhaft geführter Aufklärungsgespräche können ein Argument sein, angehenden Ärztinnen und Ärzten früh entsprechende Informationen mit auf den Weg zu geben. Bereits in der universitären Ausbildung sollte daher das Führen eines chirurgischen Aufklärungsgespräches inklusive seiner juristischen Implikationen Teil des Curriculums sein.

### Juristische Implikationen

Juristisch steht dahinter die Frage, inwieweit medizinische Behandlungen – und hier insbesondere das Aufklärungsgespräch – Studierenden zu Ausbildungszwecken übertragen werden können. Ausgangspunkt dafür ist die Feststellung, dass die medizinische Behandlung grundsätzlich von Ärzten in persönlicher Leistung erbracht wird, §630b BGB i. V. m. § 613 Abs. 1 S. 1 BGB. Danach soll die Behandlung durch den Arzt die Regel sein; dies bedeutet, dass es Ausnahmen gibt. Eine Delegation^3^ ärztlicher Leistungen ist also möglich.^4^ Besondere Schwierigkeiten bereitet dabei die Frage, welche Leistungen inhaltlich einer Delegation an nichtärztliches Personal zugänglich sind. Als allgemeine Meinung dürfte gelten, dass ein bestimmtes Spektrum ärztlicher Leistungen von der Delegationsfähigkeit ausgenommen ist, mithin also ein gesicherter „Kernbereich“ ärztlicher Tätigkeit besteht, der ausschließlich ärztlicher Verantwortung obliegt und damit nicht delegierbar ist.^5^

Die Patientenaufklärung – konkret in der Form der Selbstbestimmungsaufklärung – dient nach hergebrachter Rechtsprechung dazu, dem Patienten Art, Bedeutung und Folgen des Eingriffs („im Großen und Ganzen“^6^) bewusst zu machen, um ihn in die Lage zu versetzen, eine Entscheidung für oder gegen den Eingriff in Form einer informierten Einwilligung oder Nichteinwilligung zu treffen.^7^ Voraussetzung für eine fachlich korrekte Information über mögliche Konsequenzen der Behandlung ist demnach aber ein entsprechender medizinischer Kenntnisstand. In der Konsequenz verneint die herrschende Meinung die Delegationsfähigkeit der Aufklärung an nichtärztliche Mitarbeiter.^8^ Dieses, in seinem Kern auf alter Rechtslage fußende Meinungsbild hat auch durch Inkrafttreten des §630e BGB im Rahmen des Patientenrechtegesetztes keine maßgebliche Änderung^9^ erfahren.^10^

Nicht abschließend geklärt ist hingegen, ob diese für nichtärztliches Personal geltenden Maßstäbe auch auf Studierende im praktischen Jahr (PJler) übertragbar sind. Der Grund ist naheliegend: Studenten im praktischen Jahr bewegen sich ausbildungsgemäß an der Grenze von nichtärztlicher zu ärztlicher Tätigkeit. Dabei haben PJler zwar nach Maßgabe von §2 Abs. 1 BOÄ keine Befugnis, den Arztberuf auszuüben, erfüllen mithin also nicht die Anforderungen eines Arztvorbehaltes. Gleichzeitig sollen sie aber – denn dazu dient das praktische Jahr – unter Aufsicht des ausbildenden Arztes „ärztliche Verrichtungen durchführen“, §3 Abs. 4 S. 3 ÄApprO. Die vereinzelten, dazu ergangenen Entscheidungen tendieren zwar zugunsten einer Delegierbarkeit ärztlicher Aufklärungspflichten auf Studierende im PJ.^11^ Die Literatur lehnt dies jedoch mehrheitlich ab.^12^

In der Praxis bietet sich allerdings noch ein weiterer, wenn man so will „vermittelnder“ Weg an: Unbeschadet des Vorstehenden dürfte eine unter ärztlicher Anwesenheit und Aufsicht durchgeführte Aufklärung durch einen PJler im Einklang mit den Bestimmungen der ÄApprO nämlich unter bestimmten Voraussetzungen zulässig sein.^13^ Die durch den PJler übernommenen Aufklärungsinhalte macht sich der anwesende Arzt, soweit er nicht korrigierend eingreift, als Aufklärung zu eigen. Durch die jederzeitige Interventionsmöglichkeit ist die Qualität der Aufklärung gesichert. Tatsächlich dürfte sogar zu erwarten sein, dass sich die Qualität der Ausbildung verbessert, da einerseits der ausbildende Arzt in besonderer Weise den Empfängerhorizont bemühen muss, andererseits auch die Studierenden ein unmittelbares Verständnisfeedback der durch den Arzt praktizierten Aufklärungsinhalte ermöglichen. Da die Delegation im Kontext arbeitsteiligen Zusammenwirkens eher auf eine Verlagerung von Aufgabenbereichen zu Kapazitätszwecken zielt, dürfte es sich terminologisch bei diesem Vorgehen indes nicht mehr um Delegation im engeren Sinne handeln. Es ließe sich – anknüpfend an die im Vergleich zur Delegation einschneidendere Substitution^14^ – vom Dreiklang „Substitution – Delegation – Co-Aktion“ sprechen, in absteigender Intensität der Verlagerung von Aufgabenbereichen. Die so gelebte Co-Aktion stillt ein praktisches Bedürfnis und ein didaktisches Dilemma: Vor Beendigung der (theoretischen) Ausbildung ist es den Studierenden untersagt, Praxiserfahrung zu erwerben, während mit Approbation eben jene nicht erwerbbare Praxiserfahrung postwendend vorausgesetzt wird [12]. Stets zu berücksichtigen sind die Umstände des Einzelfalles. Neben der individuellen Eignung der Studierenden hängt die Zulässigkeit einer Aufklärung unter Aufsicht von der Art und Komplexität des Eingriffes ab, über dessen Risiken aufzuklären ist. Hier dürfte es bei solchen Eingriffen, die nicht als „Routineeingriffe“ ein typisches Risikoprofil aufweisen, eher fernliegend sein, die Aufklärung „co-aktiv“ mit einem Studierenden im praktischen Jahr durchzuführen.

Als Limitation der vorliegenden Studie ist die Beschränkung auf Berliner Studierende zu sehen, die ihre Begründung in der verlässlichen Verfügbarkeit der Kennzahlen und Ergebnisse bei gleichzeitig hoher Teilnehmerzahl hat (in Berlin ist die Teilnahme am PTM verpflichtend). Des Weiteren konnten die Fragen bislang nur jeweils einmal im PTM platziert werden, sodass differenziertere Aussagen zur Trennschärfe noch nicht möglich waren.

## Schlussfolgerung

Die vorliegende Arbeit macht deutlich, dass unter Medizinstudierenden über alle Semester hinweg deutliche Unsicherheiten bezüglich der rechtlichen Aspekte zur Durchführung eines Aufklärungsgespräches bestehen. Entsprechende Inhalte sollten vermehrt in die universitäre Lehre aufgenommen werden. Eine Möglichkeit, Studierende bereits vor Erwerb der Approbation mit dem Thema „Aufklärungsgespräch“ in all seinen Facetten vertraut zu machen und ihnen praktische Erfahrungen zu vermitteln, ist nach eingehender Analyse der juristischen Hintergründe die „Co-Aktion“: Studierende führen in Anwesenheit und unter Aufsicht des behandelnden Arztes ein Aufklärungsgespräch selbstständig durch. Nichtsdestotrotz befreit die „Co-Aktion“ den Arzt nicht von seiner Verantwortung für die Durchführung der Behandlung. Der behandelnde Arzt bleibt letztverantwortlich für die Aufklärung und muss deren ordnungsgemäße Ausführung überwachen.^15^ Die „Co-Aktion“ speziell zur Vermittlung des chirurgischen Aufklärungsgespräches wäre weniger aufwändig als beispielsweise Formate mit Simulationspatienten, sodass die Hoffnung besteht, dass sie auch im Blockpraktikum oder PJ selbst bei hoher klinischer Arbeitsbelastung der Dozierenden akzeptiert und angewendet wird. Nun gilt es zu eruieren, ob und in welcher Form die „Co-Aktion“ mit aufwändigeren Lehrformaten bezüglich der Vermittlung des Lernzieles „ein Aufklärungsgespräch führen“ vergleichbar ist. Weitere Untersuchungen zur praktischen Durchführbarkeit und Akzeptanz bei Lehrenden und Lernenden sowie Patienten sind in Planung.

## Abkürzungen

ÄApprO: Ärztliche Approbationsordnung
AG: Arbeitsgemeinschaft
BGB: Bürgerliches Gesetzbuch
BGH: Bundesgerichtshof
DGOU: Deutsche Gesellschaft für Orthopädie und Unfallchirurgie
IC: Informed Consent
KIT: Kommunikation-Interaktion-Teamtraining
MC: Multiple Choice
NKLM: Nationaler Kompetenzbasierter Lernzielkatalog Medizin
PJ: Praktisches Jahr
PTM: Progress Test Medizin
SS: Sommersemester
WS: Wintersemester

## Footnotes

1 Statt vieler: BGH, Urt. v. 08.01.1985 – VI ZR 15/83, juris, Rn. 14 = NJW 1985, 1399 (1400).

2 so auch BT-Drs. 17/10488, S. 25.

3 Bei der Delegation bleibt der Arzt letztverantwortlich für die ordnungsgemäße Ausführung; insoweit die Unterscheidung zur Substitution ärztlicher Leistungen, bei der die Verantwortung nicht mehr beim Arzt liegt, vgl. §15 Abs. 1 S. 1 SGB V i. V. m. §63 Abs. 3c SGB V i. V. m. §2 Abs. 2 S. 1 HeilkundeübertragungsRL.

4 BeckOK-BGB/*Katzenmeie*r, 54. Ed. 01.05.2020, §630b BGB, Rn. 5.

5 Statt vieler: *Katzenmeier* in Laufs/Katzenmeier/Lipp, Arztrecht, 7. Auflage 2015, X. Arztfehler und Haftpflicht, Rn. 58, m. w. N. in Fn. 211; *Spickhoff*, Medizinrecht, 3. Auflage 2018, GOÄ §4, Rn. 11.

6 BGH, Urt. v. 14.03.2006 – VI ZR 279/04, juris, Rn. 13 = BGHZ 166, 336.

7 BVerfG, Beschl. v. 02.07.1979 – 2 BvR 878/74, juris; BGH, Urt. v. 09.12.1958 – VI ZR 203/57, juris.

8 BGH, Urt. v. 27.11.1973 – VI ZR 167/72, juris; Urt. v. 28.02.1984 – VI ZR 70/82, juris; *Katzenmeier* in Laufs/Katzenmeier/Lipp, Arztrecht, 7. Auflage 2015, V. Aufklärungspflicht und Einwilligung, Rn. 46; *ders./Achterfeld*, Delegation der Selbstbestimmungsaufklärung, 2016 m. w. N. in Fn. 39, 40.

9 Im Kern geht der Streit auf in der Frage, ob §630e Abs. 1 S. 1 Nr. 1 BGB „notwendige Ausbildung“ die Approbation meint.

10 Offenbar streitig: Keine Auswirkungen der neuen Rechtslage auf das bisherige Meinungsbild erkennend: *Katzenmeier/Achterfeld*, Delegation der Selbstbestimmungsaufklärung, 2016, S. 98ff.; die Frage durch das Patientenrechtegesetz hingegen als entschieden ansehend: MüKo-BGB/*Wagner,* 8. Auflage 2020, §630e, Rn. 41, 43.

11 OLG Karlsruhe, Urt. v. 29.01.2014 – 7 U 163/12, juris = VersR 2014, 710; OLG Dresden Urt. v. 31.03.2010 – 4 U 1410/09, Rn. 20, juris; OLG Stuttgart, Urt. v. 03.12.2002 – 1 U 22/02, Rn. 75, juris; letztere unter Bezug auf die mittlerweile überholte Ausbildungsphase „Arzt im Praktikum“.

12 *Katzenmeier* in Laufs/Katzenmeier/Lipp, Arztrecht, 7. Auflage 2015, V. Aufklärungspflicht und Einwilligung, Rn. 47; *ders./Achterfeld *in: FS Bergmann 2016, 89 (92 ff.); Spickhoff/*Spickhoff*, Medizinrecht, 3. Auflage 2018, §630e BGB, Rn. 4; kritisch MüKo-BGB/*Wagner,* 8. Auflage 2020, §630e, Rn. 41, 43; dagegen Spickhoff/*Greiner*, 3. Auflage 2018, § 823 BGB, Rn. 283.

13 So wohl auch *Wienke/Sailer*, Laryngo-Rhino-Otologie 2014, 686 (687); *Häser*, Medizin im Krankenhaus, 2014, 176 (177); die Anwesenheit des Arztes hingegen nicht für notwendig erachtend OLG Karlsruhe, Urt. v. 29.01.2014 – 7 U 163/12, juris = VersR 2014, 710; *Makoski*, jurisPR-MedizinR 3/2014, Anm. 1; jedenfalls die Aufklärung unter Anwesenheit des ausbildenden Artes nicht als per se unzulässige Delegation wertend BeckOK-BGB/*Katzenmeier*, 54. Ed. 01.05.2020, §630e, Rn. 38.

14 Vgl. oben, Fn. 3.

15 BGH, Urt. v. 07.11.2006, VI ZR 206/05, juris; Spickhoff/*Greiner*, 3. Auflage 2018, § 823 BGB, Rn. 283.
